# Thermal management with innovative fibers and textiles: manipulating heat transport, storage and conversion

**DOI:** 10.1093/nsr/nwae295

**Published:** 2024-08-22

**Authors:** Yucan Peng, Yi Cui

**Affiliations:** Department of Energy and Resources Engineering, College of Engineering, Peking University, Beijing 100871, China; Department of Materials Science and Engineering, Stanford University, Stanford, CA 94305, USA; Department of Energy Science and Engineering, Stanford University, Stanford, CA 94305, USA; Stanford Institute for Materials and Energy Sciences, SLAC National Accelerator Laboratory, Menlo Park, CA 94025, USA

**Keywords:** thermal management, fibers and textiles, heat transport, heat storage, energy conversion

## Abstract

Thermal management is essential for maintaining optimal performance across various applications, including personal comfort, electronic systems and industrial processes. Thermal-management fibers and textiles have emerged as innovative solutions to manipulate heat transport, storage and conversion efficiently. This review explores recent advancements in material innovations in this field. We summarize the novel fibers and textiles designed for controlling heat transport through different pathways, progress in developing phase-change-material-based fibers and textiles for heat storage regulation, and application of photothermal conversion, Joule heating and thermoelectric effect as energy conversion routes in advanced fibers and textiles. Furthermore, we discuss the challenges and future perspectives of this field. It is believed that ongoing research and development promise to bring about innovative thermal-management solutions catering to demands across multiple sectors.

## Teaser

This review highlights recent innovations in thermal management fibers and textiles, focusing on advances in heat transport, storage, and energy conversion, while discussing the challenges and future perspectives of this evolving field.

## INTRODUCTION

Thermal management is critical in a wide range of applications, encompassing everything from personal comfort in clothing to the energy performance of buildings and efficiency of electronic devices [1–3]. Effective thermal management ensures that the objects of interest maintain optimal temperatures, thereby enhancing their performance and efficiency. For instance, in wearable technology, athletes require garments that can ideally regulate body temperature and moisture transfer, thus allowing them to maximize their potential [4]. In building construction and retrofit, advanced thermal-management materials are essential for improving energy efficiency, reducing heating and cooling costs, and contributing to more sustainable living environments [5]. Similarly, in the realm of electronics, as devices are becoming smaller and more powerful, efficient thermal management is crucial to prevent overheating, which leads to reduced performance and lifespan. In the context of global warming and the continuous progress of technological advancements [6], the demand for innovative thermal-management solutions has never been more significant.

In the past, traditional materials in assorted categories have been widely used for thermal management, including metals, ceramics and polymers [7–9]. People take advantage of their intrinsic thermal properties for applications in certain scenarios [10]. For example, cotton and wool have relatively low thermal conductivity so they are usually used for heat insulation [11]. However, traditional materials cannot fully satisfy the rapidly growing demand of thermal management. Their limitations, and the emerging needs in various fields, drive the search for novel materials that can offer enhanced thermal-management capabilities, shifting towards improved manipulation of heat transport, storage and conversion.

Heat is a form of energy that is transferred between systems or objects with different temperatures. The fundamental laws of heat, also known as the laws of thermodynamics, describe the principles governing the behavior of thermal energy. The laws of heat transfer describe the movement process of heat between different objects/systems, such as Fourier's law of heat conduction and Stefan-Boltzmann's law. Thermal properties of materials, including thermal conductivity, specific heat capacity and latent heat, define materials’ performance and their features with regard to interacting with and manipulating heat. The design and development of innovative thermal-management materials adhere to these basic principles and meet specific application requirements by achieving enhanced thermal properties.

Innovative fibers and textiles, among thermal-management materials, represent a frontier in this area and they are emerging as promising candidates for effective thermal management [12]. The unique advantages of being flexible, lightweight and versatile make them ideal for a wide range of applications, from advanced wearables to building materials [13]. Their ability to be woven, knitted or non-woven into various structures allows for the creation of customized thermal-management solutions that can meet the specific needs of different fields [14,15]. Researchers and engineers have been designing and developing novel fibers and textiles, enabling tailored thermal-management solutions via constructing materials at the micro- and nanoscale to achieve precise control over thermal properties.

In the commercial market, advancements in fiber and textile technology have led to the commercialization of some thermal-management products. They are specifically designed to regulate temperature and enhance comfort, integrated into brands such as Omni-heat, Outlast, Gore-Tex, CoolMax and Coolcore, catering to diverse applications such as sportswear, healthcare and military gear. Some techniques exploit special fiber shapes for enhanced moisture and heat transport, like CoolMax, which employs unique four-channel shape fibers. Some develop progressive finishing processes to add functional materials onto traditional fabrics, such as Omni-heat, which utilizes heat-reflective patterns inside the garment to reduce body heat loss. Additionally, brands like Gore-Tex have developed advanced expanded polytetrafluoroethylene (ePTFE) membrane technology so that they block external water and wind, enhancing overall thermal regulation while expelling sweat vapor for breathability. Coolcore fabrics utilize moisture-wicking technologies without the use of chemicals, which can draw sweat away from the skin and allow it to evaporate quickly. Another notable example is Outlast, which incorporates phase change materials (PCMs) into fibers that absorb, store and release heat depending on the surrounding temperature, thus maintaining optimal thermal comfort for the wearer. The commercial success of these fibers and textiles is largely attributed to the significant demand for superior thermal management. At the same time, they are pushing the boundaries of what is possible in modern apparel technology and also spurring research into more cutting-edge materials in the academic community.

**Figure 1.**
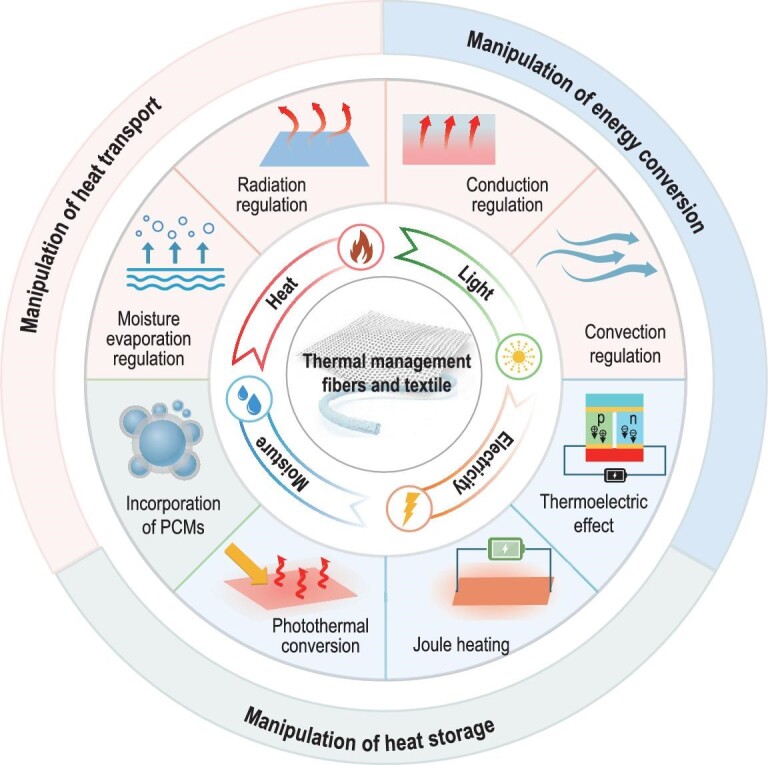
Schematic overview of the innovative thermal-management fibers and textiles discussed in this review. The material innovations encompassing manipulation of heat transport, heat storage and energy conversion are highlighted.

This review is structured to provide a thorough exploration of innovative fibers and textiles for thermal management, covering the latest research in the field (Fig. 1). We categorize novel thermal-management fibers/textiles into three types based on their interaction with and influence on heat: materials that manipulate heat transport, store heat, and convert or generate heat. The first section offers an in-depth overview of fibers/textiles that facilitate or suppress heat transport through radiation, conduction, convection and moisture evaporation. The second section introduces novel fibers/textiles incorporating heat storage functionality. The third section discusses fibers/textiles participating in energy conversion involving thermal energy. Passive, active and dynamic responsive materials will all be highlighted. We will review the design insights, fabrication techniques, thermal-management mechanisms and application scenarios, as well as their respective advantages and limitations. This review aims to provide a comprehensive understanding of the current progress in thermal-management fibers and textiles, highlighting both the opportunities and challenges in this rapidly evolving field.

## MANIPULATION OF HEAT TRANSPORT

Heat transport is a critical aspect in thermal management, concerning the movement of heat from one location to another through mechanisms including radiation, conduction, convection and evaporation, each having specific applications in fibers and textiles. Innovative fibers and textiles that manipulate radiation, conduction, convection and evaporation are discussed in this section, showcasing their role in meeting functional needs.

### Fibers and textiles for radiation regulation

Thermal radiation, a form of electromagnetic radiation, is emitted by all objects based on their temperature. It plays a crucial role in various applications, from heating and cooling systems to space technology. In the context of fibers and textiles, regulating thermal radiation is essential for developing materials that provide effective thermal management, ensuring comfort and protection in diverse environments. This review focuses on thermal radiation regulation using fibers and textiles, specifically targeting the mid-infrared (MIR, 7–14 μm) and solar spectrum, including the visible (400–700 nm) and near-infrared (NIR, 0.7–2.5 μm) wavelength ranges, deliberately excluding electromagnetic shielding from its scope. The latter, while related to radiation management, involves different principles and materials that are outside the purview of this discussion.

The ability of materials to regulate thermal radiation heavily depends on their surface optical properties, including emissivity, reflectivity and transmissivity [16]. In other words, designing and developing novel fibers and textiles for radiation regulation means tuning their optical properties through strategies such as molecular design [17], surface modification [18] and microstructure construction [19]. The fundamental principle of passive thermal management is to maximize heat dissipation for cooling purposes while minimizing heat loss to retain warmth.

#### MIR transparent fibers/textiles for radiative cooling

**Figure 2.**
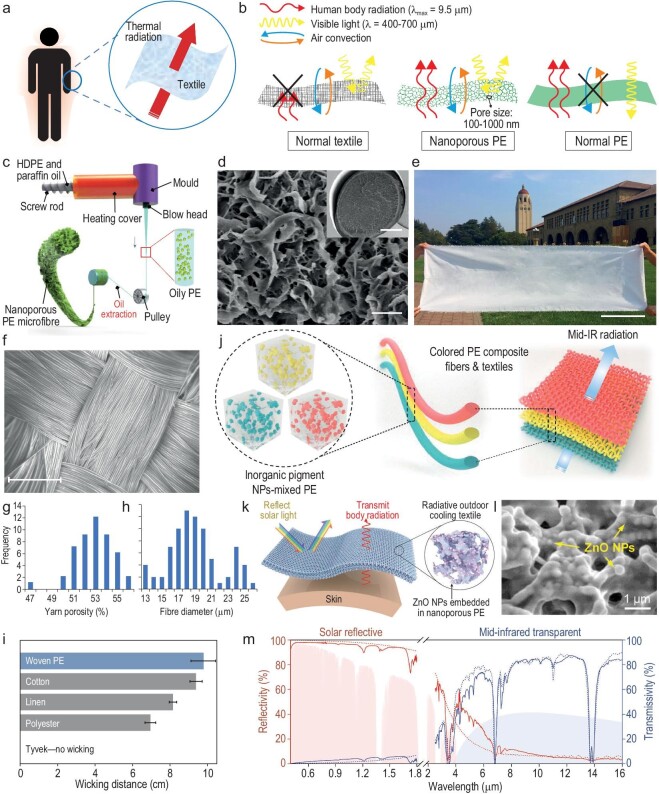
MIR transparent fibers and textiles based on PE for radiative cooling. (a) Schematic illustrating the design principle of MIR-transparent textiles for human-body thermal-radiation dissipation. (b) Schematics of the comparison between NanoPE, normal PE and cotton. Reproduced with permission from [17]. Copyright 2016, AAAS. (c) A schematic diagram of the manufacturing process for NanoPE fibers. (d) Scanning electron microscope (SEM) image of the cross-section view of a NanoPE fiber. Scale bar, 2 μm. The inset shows a lower-magnification SEM image of the well-preserved cross-section of the fiber. Scale bar, 50 μm. (e) A photograph of a large woven NanoPE fabric. Scale bar, 0.35 m. (c–e) reproduced with permission from [19]. Copyright 2018, Nature Publishing Group. (f) SEM top-view image of the water-wicking woven PE fabric. Scale bar, 500 μm. (g–h) Frequency distributions of yarn porosity (g) and fiber diameter (h) evaluated on 48 cross-sectional yarn images and 100 individual fiber images, respectively, based on SEM and micro computed tomography (micro-CT) analysis. (i) Vertical wicking distances in the water-wicking woven PE fabric (blue bar) and in commercial woven textiles (gray bars) 10 min after contact with water. (f–i) reproduced with permission from [23]. Copyright 2018, Nature Publishing Group. (j) Design schematic for the coloration of radiative cooling textiles. Reproduced with permission from [21]. Copyright 2019, Elsevier B.V. (k) Schematic of the ZnO NPs–embedded NanoPE textile, designed for radiative outdoor cooling by reflecting sunlight and transmitting human-body thermal radiation. (l) High-magnification SEM image showing the morphology of ZnO NPs distributed in a NanoPE matrix. (m) Measured (solid lines) and simulated (dashed lines) reflectivity and transmissivity spectra of ZnO-PE from ultraviolet to mid-infrared range (0.3–16 μm). Reproduced with permission from [22]. Copyright 2018, Wiley-VCH GmbH.

MIR transparent fibers and textiles are designed to allow MIR radiation to pass through them, reducing heat buildup and promoting cooling (Fig. 2a) [17,19–23]. Intrinsic MIR transparent materials are not common, since the MIR spectrum overlaps with most of the IR absorption wavelengths of common textile materials, such as C−O stretching, C−N stretching and aromatic C−H bending [17]. On the other hand, polyolefins such as polyethylene (PE) are intrinsically transparent in the MIR wavelength range. This is due to their simple structure, which consists solely of aliphatic C−C and C−H bonds, resulting in high transparency in the MIR range (7–14 μm) with only a few narrow absorption peaks [17]. However, to be suitable for specific applications, other properties of PE must be modified appropriately. For instance, the inherent visible transparency of PE lacking shielding functions, and its mechanical rigidity, inhibit its suitable use for personal garments.

Aiming at applying PE to personal radiative cooling, Tong *et al*. theoretically proposed an IR-transparent visibly opaque fabric (ITVOF) [20]. In their conception, synthetic PE fibers, which intrinsically absorb very little IR radiation, are structured to provide visible opacity by strong Mie scattering, thereby achieving high transmittance in the MIR region and decent reflectance across visible wavelengths. Hsu *et al*. demonstrated nanoporous polyethylene (NanoPE) as a novel candidate material for human body radiative cooling [17]. Nanoscale pores of 50–1000 nm are embedded in the PE film to strongly scatter visible light, while the mismatch with MIR wavelength range ensures that the MIR transparency is not destroyed (Fig. 2b) [17]. Furthermore, Peng *et al*. developed a large-scale extrusion technique for uniform and continuous NanoPE fibers (Fig. 2c and d) and first fabricated the NanoPE fabric using industrial manufacturing processes (Fig. 2e) [19]. The NanoPE fabric exhibits ∼80% MIR transmittance, nearly 90% visible opacity and decent wearability. It can achieve a cooling effect of 2.3°C compared to conventional cotton fabric of similar thickness, corresponding to ∼20.1% indoor cooling energy savings [19]. To endow PE-based textiles with facilitated moisture-transport performance, Alberghini *et al.* engineered PE fibers, yarns and fabrics to achieve efficient water wicking and fast-drying performance [23]. They carefully optimize the water-fiber contact angle, fiber radius, yarn structure and yarn porosity in a multifilament yarn that is the building block in the fabric (Fig. 2f–h). The water-wicking properties of the woven PE fabric even exceed those of natural (cotton and linen) and synthetic (polyester) commercial textiles, as shown in Fig. 2i [23].

Based on PE as an MIR transparent material, Cai *et al*. employed IR-transparent inorganic nanoparticles (NPs) as pigments for PE and reported scalable colored PE fibers and fabrics, as illustrated in Fig. 2j, which can realize a ∼2°C cooling effect as well [21]. By incorporating zinc oxide (ZnO) NPs into NanoPE, a spectrally selective nanocomposite textile (ZnO-PE) was demonstrated for outdoor personal radiative cooling (Fig. 2k and l) [22]. It allows the transmission of human body thermal radiation into the cold sky (nearly 80% transmittance), while reflecting solar heat (>90%) to realize an enhanced cooling performance (Fig. 2m) [22]. In addition, researchers are combining PE with other traditional textile materials to fabricate composite textiles, aiming at achieving a balance between good wearability and MIR transparency.

Apart from relying on intrinsic MIR transparent materials like PE, researchers have been proposing photonic structure design approaches to realize decent MIR transparency through blending IR-opaque fibers and largely IR-transparent fibers. For example, Catrysse *et al*. theoretically designed a photonic structure textile based on cotton and nylon fibers [24]. Their calculations show promising results in that the photonic structure textile containing cotton and nylon can achieve a 2.2°C cooling effect compared with cotton-only textiles.

#### Fibers/textiles with high MIR emissivity for radiative cooling

In contrast to MIR transparent materials that transmit thermal radiation, MIR-emissive fibers and textiles are engineered to efficiently emit thermal radiation for heat dissipation, offering different yet complementary approaches to thermal management. Intrinsic MIR absorptive (i.e. MIR-emissive) materials are much more common than MIR transparent materials. Many types of materials, including polymers, ceramics and carbon-based materials, typically exhibit high emissivity in the MIR wavelength range [25]. These materials have been utilized to develop various radiative cooling solutions, extending beyond just fibers and textiles [26]. Within the scope of this review, we will discuss a selection of significant research efforts in this field.

**Figure 3.**
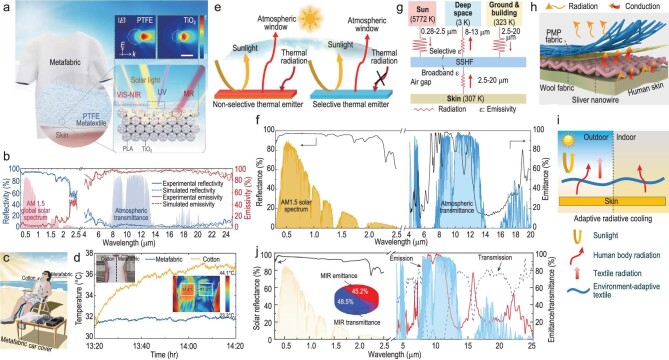
Textiles with high MIR emissivity for radiative cooling. (a) Schematic of the hierarchical morphology metafabric for daytime radiative cooling. (b) Measured reflectivity and emissivity spectra of the metafabric (0.3 to 25 µm). (c) Schematic of the metafabric cooling tests on the human body. (d) Temperature tracking for skin under different fabrics in direct sunlight in Guangzhou, China (23°5′32′′N, 113°23′45′′E, 7 December 2020). The insets show photographs and thermal images of the volunteer wearing a homemade vest. (a–d) reproduced with permission from [28]. Copyright 2021, AAAS. (e) Schematic of the radiative heat transfer process of a selective and non-selective thermal emitter. The selective thermal emitter has suppressed absorption of thermal radiation from the surroundings compared to that of the non-selective emitter. (f) UV–visible–infrared reflectance/emittance of a 500 μm es-PEO film (black line). (e and f) reproduced with permission from [30]. Copyright 2021, Nature Publishing Group. (g) Radiative heat transfer network of SSHF in outdoor environments. For simplicity, the convective and conductive heat transfer are not shown. (h) Structure of the multilevel SSHF composed of PMP fabric, AgNW and a wool fabric layer. (g and h) reproduced with permission from [31]. Copyright 2024, AAAS. (i) Schematic of the textile design that features all-weather adaptive human-body radiative cooling. (j) Spectral response of an ∼260-μm-thick POM textile in the 0.3–25 μm wavelength range, including solar reflectance (black line), MIR emittance (red line) and MIR transmittance (blue line). Inset: the ratio of emittance, transmittance and reflectance in the MIR region. (i and j) reproduced with permission from [32]. Copyright 2023, Nature Publishing Group.

Hsu *et al*. reported a dual-mode textile with different emissivity of its two surfaces for radiative cooling and warming [27]. The high-emissivity surface can lead to a 3.1°C cooling effect in an indoor test, which utilizes 9 μm carbon to achieve ∼0.9 emissivity [27]. For outdoor scenarios, the atmospheric MIR transparency window crossing 8–13 μm can be utilized for dissipating heat into cold sky, and the factor of solar heating should be considered. As shown in Fig. 3a, Zeng *et al.* fabricated a hierarchical-morphology metafabric, which provides 92.4% reflectance in the solar spectrum and high emissivity (94.5%) in the atmospheric window, effectively resisting ultraviolet (UV), visible light and NIR light, while efficiently emitting heat in the MIR region (Fig. 3b) [28]. They composed a homemade vest by sewing a commercial cotton fabric and a metafabric together. It was worn by a volunteer who reclined under direct sunlight for an hour, to validate the cooling performance with scalable and wearable features in pragmatic scenarios, as illustrated in Fig. 3c. The IR images displayed a large temperature difference between the two sides (34.4°C and 31°C). Thermocouples adhered under the vest also indicated a cooling effect of ∼4.8°C offered by the metafabric (Fig. 3d) [28]. Zhu *et al*. explored the nanoprocessing of silk through a molecular bonding design and scalable coupling reagent-assisted dip-coating method to overcome its intrinsic absorption of protein in the UV region, and demonstrated that the processed silk can achieve daytime radiative cooling [29].

In addition to wisely engineering material to be highly emissive in the 8–13 μm atmospheric transparency window (ATW) for efficiently emitting heat, the optical property outside this region should be deliberately designed as well. As illustrated in Fig. 3e, Li *et al*. fabricated a hierarchically designed polymer nanofiber-based film, i.e. electrostatic spinning polyethylene oxide (es-PEO), by a scalable electrostatic spinning process [30]. The es-PEO has selective mid-infrared emission enabled. The C−O−C (1260–1110 cm^−1^) and C−OH (1239–1030 cm^−1^) bonding endows the es-PEO with a selective emissivity of 78% in the 8–13 μm wavelength range (Fig. 3f). Compared to a non-selective emitter, the authors observed ∼3°C cooling improvement at night [30]. Recently, Wu *et al*. developed an MIR spectrally selective hierarchical fabric (SSHF) with emissivity greatly dominant in the atmospheric transmission window, to minimize the net heat gain from the surroundings (Fig. 3g) [31]. The SSHF was engineered by laminating polymethylpentene (PMP), silver nanowires (AgNWs) and a wool fabric layer into a multilevel fabric, as depicted in Fig. 3h, in which PMP achieved outward-facing emissivity in ATW, AgNWs suppressed the outward-facing non-ATW emissions, and wool ensured the inward-facing broadband emissivity [31].

Wu and the authors conceived an all-weather radiative human body cooling textile, polyoxymethylene (POM) nanotextile (Fig. 3i), which not only achieves selective emission in the atmospheric window (8–13 μm) but also shows transmission in the remaining mid-infrared wavebands as well as reflection of sunlight (0.3–2.5 μm) [32], as exhibited in Fig. 3j. They compared the POM textile with typical transmission-type, emission-type and commercial cotton textiles under sunny outdoor, cloudy outdoor and indoor environments. Due to the special spectrum design, the POM textile exhibits an enhanced cooling performance. These novel design strategies for radiative cooling textiles are worthy of reference [32].

#### MIR reflective fibers/textiles for radiative warming

In contrast to materials designed to be cooling, radiative warming demands a drastic reduction of heat loss so that materials should be able to reflect MIR radiation (low emissivity). To conceal the intrinsic high MIR emissivity feature of common textile materials, low-emissivity (low-e) materials, such as metal, are usually employed to modify the surface radiative property. A metallic AgNW-embedded cloth was demonstrated for personal thermal management [33]. The authors aimed to form a conductive network to reflect human body MIR radiation, and supply Joule heating to complement the passive insulation. The AgNW cloth shows 40.8% reflectance in MIR wavelength range, exhibiting more enhanced reflectivity than the normal cloth (1.3% reflectance) owing to the modification of AgNW [33]. Peng *et al.* reported a bifunctional asymmetric fabric (BAF) that utilized Ag electroless plating to conformally coat Ag onto every fiber within the fabric. The low-e side of BAF presents ∼70% reflectance in the MIR region. In the warming mode, it serves as a low-e surface facing out, increasing the radiative heat transfer resistance [34].

**Figure 4.**
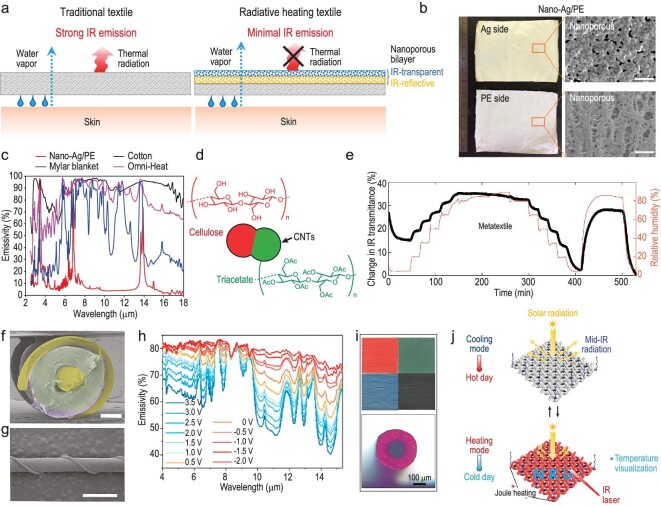
MIR-reflective textiles for radiative warming and responsive fibers/textiles for dynamic radiation regulation. (a) Schematics depicting the heat dissipation and vapor transmission of a human body covered with a traditional textile and a Nano-Ag/PE textile. (b) Photos and SEM images of the Ag side and PE side of nano-Ag/PE. Scale bar, 1 μm. (c) Measured total Fourier transform infrared spectrometer emittance at the textile outer surface of nano-Ag/PE, cotton, Mylar blanket and Omni-Heat. (a–c) reproduced with permission from [18]. Copyright 2017, Nature Publishing Group. (d) A metafiber design based on CNT-coated triacetate-cellulose side-by-side bimorph fibers. (e) IR gating of the metatextile (black line) at different relative humidity profiles. (d and e) reproduced with permission from [38]. Copyright 2019, AAAS. (f and g) Cross-sectional (f) and surface (g) SEM images of radiative electrochromic fibers. Scale bars: (f) 200 μm and (g) 1 mm. (h) IR emissivity of radiative electrochromic fibers at various voltages. (f–h) reproduced with permission from [39]. Copyright 2024, Wiley-VCH GmbH. (i) Optical (left) and micrograph (right) images of the TC conductive fiber. (j) Working mechanism of personal thermal management and temperature visualization of fabric woven by TC conductive fibers in hot (left) and cold (right) conditions. (i and j) reproduced with permission from [42]. Copyright 2023, American Chemical Society.

Cai *et al*. demonstrated a nanophotonic structure textile (Nano-Ag/PE) with superior passive radiative personal warming capability and no sacrifice of wearability [18], as elucidated in Fig. 4a. They employed the Ag electroless plating process to deposit Ag film with nanoscale pores onto NanoPE film, then laminated with a cotton fabric. The interconnected nanoporous metallized PE achieves a minimal IR emissivity of 10.1% on the non-metallized PE side (Fig. 4b and c), and in the meantime the nanopores provide a pathway for the transmission of water vapor. Laminated on the outer surface of traditional cotton textile, the Nano-Ag/PE can enable a 7.1°C decrease in the set-point of ambient temperature compared to traditional cotton textile, greatly outperforming other radiative warming textiles, such as Omni-Heat (0.1°C) and Mylar blankets (4°C) [18]. Notably, they also performed heat transfer model analysis and experiments to reveal the necessity of low-IR emissivity on the textile outer surface, rather than the inner surface, providing insightful guidelines for radiative warming textiles [18].

One of the most direct applications of MIR reflective textiles is in outdoor apparel, particularly in high-altitude climbing gear. These textiles help climbers maintain a stable body temperature under extreme weather conditions. Their lightweight properties, breathability and significant improvement in thermal comfort not only reduce the risk of heat-related stress but also enhance safety and the endurance of users.

#### Fibers/textiles with dynamic radiation properties

Compared to fibers/textiles with changeless radiative features, the ones endowed with dynamic properties are promising for adaptive radiation regulation, offering more flexibility. The dual-mode textile reported by Hsu *et al.*, as mentioned above, provides cooling and warming modes. The textile can be flipped so that either high emissivity or low emissivity is on the outside [27]. The bifunctional asymmetric fabric with tailored radiation and conduction utilizes a similar emissivity design in that two surfaces of the textile display distinct emissivity for facilitating heat dissipation or reducing heat loss [34]. The radiation property changes of these textiles, requiring flip-over operations, depend on the users.

Stimuli-responsive IR modulator devices, based on signals such as humidity, temperature, electricity and mechanical deformation, have been widely reported [35–37]. Research into textiles made of fibers/yarns featuring dynamic radiation properties is also emerging [38–42]. For example, Zhang *et al.* fabricated triacetate-cellulose biomorphic fibers (metafibers) whose two components display different responses to moisture [38]. The fibers are coated with a thin layer of carbon nanotubes (CNTs) to make them electrically conductive, then the fibers serve as elements for controlling distance-dependent electromagnetic interactions, as portrayed in Fig. 4d. Such a design allows the textile made of metafibers to be IR adaptive, responding to the relative humidity changes. A more than 35% change in MIR transmittance is realized by the fabric woven by these metafibers (Fig. 4e) [38]. This work pioneered a new approach for developing IR-transmittance responsive textiles. However, it did not include a durability evaluation, which should be regarded as an essential performance metric in future studies.

A dynamic thermoregulatory textile woven from scalable-manufactured radiative electrochromic fibers was fabricated by Fan and co-authors [39]. A CNT dispersion, electrolyte slurry and CNT dispersion were sequentially coated onto a Cu@Ni metal wire, and finally a spiral twined metal wire was added as the outer electrode (Fig. 4f and g). The IR emission of the outer CNT layer was controlled by the electrochemical doping of ions on the radiative electrochromic fibers. A modulated emissivity of Δε ≈ 0.35 (Fig. 4h) and excellent electrochemical controllability of over 100 m in length within 5 seconds were achieved when applying voltage switch between -2 and 3.5 V [39]. To improve durability, a thin IR-transparent PE layer was coated onto the electrochromic fibers through continuous extrusion, which achieved good stability (100 washing cycles and 5000 abrasion cycles). Moreover, Liu *et al*. reported a multiband spectral regulation fiber exhibiting Δε = 0.528 within the 8–14 µm, low applied potentials (−0.8 V and 0.8 V), fast response time (2.44 s) and robust long-term cycling stability (over 5000 cycles), based on the redox reaction of polyaniline (PANI) [40].

In addition to the IR wavelength range, thermochromic (TC) and electrochromic (EC) fibers and textiles that show responsive color switching in the solar spectrum are exploited. Yu *et al*. selected four types of TC microparticles and fabricated TC conductive fibers with a coaxial structure composed of conductive cores and a TC shell [42], as shown in Fig. 4i. In the cold, the colorful appearance brings about more solar absorption and photothermal effect. In hot conditions, the TC conductive fabric turns white, resisting solar heating and exhibiting a maximum temperature drop of 2.5°C compared to commercial white fabrics (Fig. 4j) [42]. Employing a piece of nylon fabric as the substrate, Fu *et al*. plated gold as the conducting layer, deposited EC-active PANI and aluminum coatings, and filled the dense micropores with semi-solid gel electrolyte, demonstrating a highly integrated all-in-one EC fabric [41]. The applied voltage variation from -0.4 to 0.6 V can lead to an obvious blue shift of the reflection peak during the oxidation of PANI. It can not only achieve a reversible color switch between yellow and green, but also provide nearly 5°C temperature difference [41]. These dynamic radiation properties in the visible wavelength range are valuable for camouflage clothing as well. Such novel materials would be more appealing if they demonstrated sufficient durability for practical application.

### Fibers and textiles for thermal conduction regulation

Conduction involves the transfer of heat through direct contact between objects, where thermal energy moves from the hotter region to the cooler region. Thermal conductivity is a key thermal property which measures a material's ability to conduct heat. For enhanced thermal-management capabilities, fibers/textiles engineered to have a tailored thermal conduction performance are emerging. These advanced materials are designed to either dissipate heat efficiently or retain it, depending on the specific application requirements. Innovative techniques, such as incorporation of nanomaterials, construction of nanostructures and optimization of weaving/knitting patterns, have been developed to regulate the thermal conduction performance of fibers and textiles.

#### High thermal conductivity fibers/textiles

Various strategies have been applied to enhance thermal conductivity for fibers and textiles, including utilization of intrinsic high thermal conductivity materials, optimization of the internal structure of fibers, incorporation of fillers and coatings, and refining the weaving or knitting patterns [43–48]. These approaches can be applied individually or in combinations.

Materials with inherently high thermal conductivity, such as metals (silver, copper, aluminum, etc.), ceramics (boron nitride, aluminum nitride, etc.) and carbon-based materials (graphene, CNTs, etc.), are prime candidates for developing high thermal conductivity fibers. Fiber processing techniques for these materials have been developing, and these fibers can be woven or knitted into textiles either on their own or blended with other fibers [49].

**Figure 5.**
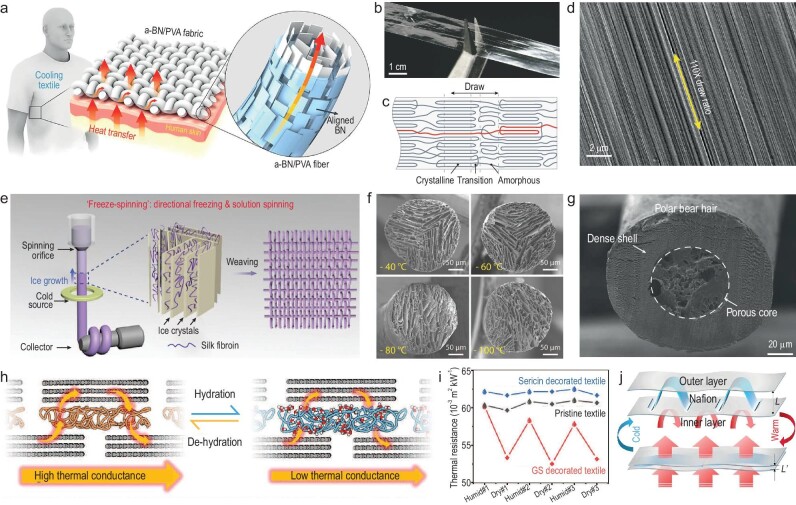
Fibers and textiles for thermal conduction regulation. (a) Schematic illustration of the a-BN/PVA textile. The highly aligned BNNSs in the microfibers act as efficient heat conduction pathways. Reproduced with permission from [43]. Copyright 2017, American Chemical Society. (b) Photo of an ultrahigh molecular weight polyethylene (UHMWPE) thin transparent drawn film. (c) Illustration of the oriented crystallites interconnected by aligned amorphous chains in the ultra-drawn PE films. (d) SEM image of a film with 110 × draw ratio. (b–d) reproduced with permission from [52]. Copyright 2019, Nature Publishing Group. (e) Schematic illustration of the ‘freeze-spinning’ technique. (f) Radial cross-sectional SEM images showing different porous structures of biomimetic fibers prepared at different freezing temperatures: −40, −60, −80 and −100°C, respectively. (e and f) reproduced with permission from [60]. Copyright 2018, Wiley-VCH GmbH. (g) SEM image of an EAF. Reproduced with permission from [61]. Copyright 2023, AAAS. (h) Illustration showing the regulation of thermal conductivity in GS, as it pertains to hydration/dehydration processes. (i) Variation of the thermal resistance of a GS-decorated textile and other textiles in cyclic humid-dry atmospheres. (h and i) reproduced with permission from [62]. Copyright 2022, Wiley-VCH GmbH. (j) Schematic showing a thickness-reversible structure using Nafion as a thermally adaptive interlayer. Reproduced with permission from [63]. Copyright 2017, Nature Publishing Group.

Another strategy for improving fiber's thermal conductivity is to incorporate these high thermal conductivity materials as fillers or coatings of conventional fibers/textiles. As demonstrated in Fig. 5a, Gao *et al*. reported a 3D printed textile made of highly aligned boron nitride nanosheets (BNNSs)/poly(vinyl alcohol) (PVA) composite (a-BN/PVA) [43]. Due to the thermally conductive and highly aligned BNNSs, the a-BN/PVA textile presents 0.078 W/m·K thermal conductivity, over twice that of the measured cotton fabric (0.035 W/m·K). In addition, by coating multi-walled carbon nanotubes (MWCNTs) onto the surface of cotton fabrics, the thermal conductivity of cotton fabrics can be increased by 1.5 times with 50% MWCNT content [50].

Apart from involving intrinsic high thermal conductivity materials to form composites, the internal structure of fibers can significantly influence their thermal conductivity as well. Common polymer materials for clothing applications usually show relatively low thermal conductivity (0.1–0.5 W/m·K). However, over a decade ago, it was reported that the thermal conductivity of ultra-drawn PE nanofibers whose polymer chains are highly oriented can reach a thermal conductivity as high as ∼104 W/m·K [51]. Later, the same research group demonstrated drawn PE films achieving a thermal conductivity of 62 W/m·K, which was scaled up from the previous individual nanofiber to the macroscale (Fig. 5b–d) [52]. Xiang *et al*. prepared polyimide (PI) fibers by wet-spinning and subsequent hot drawing treatment and investigated the effect of thermal drawing on thermal properties. Similarly, it was found that thermal drawing was effective for improving thermal conductivity and diffusivity of PI fibers. This is because increasing the crystallinity of polymer fibers can reduce phonon scattering [53].

At the fabric level, the pattern into which fibers are woven or knitted together can greatly impact the thermal conductivity of the resulting textile [54]. Optimizing the weaving/knitting patterns allows for better heat flow. For instance, patterns that maximize fiber contact and reduce air gaps within the textile structure can improve heat conduction. Advanced textile engineering methods, including 3D weaving and knitting techniques, can be employed to design and produce fabrics with optimized thermal properties.

#### Low thermal conductivity fibers/textiles

For insulation purposes aimed at minimizing heat dissipation, the main challenge is efficiently trapping small air pockets within fibers or textiles. This trapped air acts as a barrier to heat transfer, enhancing the insulating properties [55,56].

Integrating low-conductivity fillers into the fiber matrix or applying insulating coatings on the fiber surface can reduce thermal conductivity [57,58]. Fillers such as silica aerogels or polymeric microspheres are known for their excellent insulating properties. Coatings made from materials with low thermal conductivity, like aerogels, can create an additional barrier to heat transfer, improving the overall insulation of the textile [58].

Fine fibers or shape-profiled fibers are capable of creating more surface area and increasing the number of air pockets within the textile structure. One example is natural down fibers, which are extensively used in warm clothes. Thinsulate, a commercial product of 3M, contains 15-µm-diameter fibers that are much thinner than normal fibers, and thus heat flow can be effectively reduced [55]. Profiled fibers, with complex cross-section shapes, can further enhance the thermal insulation effect by creating additional voids [59].

Engineering fibers to be hollow or porous is another approach to trapping air and impeding heat flow. Cui *et al*. developed a ‘freeze-spinning’ technique to realize continuous and large-scale fabrication of fibers with an aligned porous structure, mimicking polar bear hairs, as presented in Fig. 5e [60]. The as-prepared fibers show up to 87% porosity and highly axially aligned pores (Fig. 5f). A woven textile was made from such biomimetic fibers and exhibits excellent thermal insulation performance [60]. Recently, to overcome the fragility and poor processability of aerogel materials, the same group designed an encapsulated aerogel fiber (EAF) [61]. Compared to their previous work, this study applied thermoplastic polyurethane (TPU) as the shell layer, taking advantage of its high mechanical strength and stretchability, as shown in Fig. 5g [61]. The EAF displays a similar core-shell structure to polar bear hairs. It has over 90% internal porosity while possessing a large tensile strength of around 7.3 to 12.7 MPa and super stretchability of around 1000%–1600% strain, nearly three orders of magnitude better than traditional aerogel fibers (usually around 2% strain). A sweater knitted with EAFs that is only around one-fifth as thick as down can achieve the same warmth, and good washability and dyeability [61].

#### Fibers/textiles with dynamic thermal conductivities

Fibers and textiles with dynamic thermal conductivities represent a more flexible approach to thermal management, allowing materials to adapt their thermal conductivities in response to environmental changes or specific needs. Liang *et al*. explored a humidity-driven and flexible thermal conductance regulating material, which is composed of brick-and-mortar structured graphene and silk sericin (GS) [62]. The regulation was ascribed to the hydration/dehydration of sericin and the subsequent changing in graphene-sericin interfacial thermal conductance, as explained in Fig. 5h. The GS material can achieve an impressive switching ratio up to 14 times that of thermal conductivity. Applying 400 nm thick GS onto a commercial textile guaranteed an active response of thermal resistance of the GS-decorated textile toward a variation in humidity. In a three-cycle test, the GS-decorated textile in a humid state shows a ∼10% reduction in thermal resistance compared to the dry state (Fig. 5i) [62]. Besides, some designs exhibiting air-gap size changes can result in thermal conductance alteration. In the work of Zhong *et al.*, the researchers designed thickness-adjustable clothes by inserting bent Nafion sheets between two fabrics [63]. Due to the shape change of Nafion in response to humidity, the gap between the two fabrics becomes thinner to enhance heat conductance as humidity increases. It recovers its original thickness upon humidity decrease and restores its thermal insulation (Fig. 5j) [63]. Additionally, the researchers conducted 400 open-and-close cycles to demonstrate the excellent durability and repeatability of this design [63]. Nevertheless, the design of fabrics with tunable thermal conduction has not been explored as extensively as other heat transfer mechanisms. We anticipate more research and development in this direction.

### Convective thermal regulation in fibers and textiles

Convection is another crucial heat transfer route, involving the movement of heat through fluids (such as air and liquid) around the concerned object. Effective management of convection in fibers and textiles can lead to improved temperature control and comfort [64]. Factors including material properties (specific heat capacity, density, surface characteristics, etc.), structural design elements (fiber diameter, shape, porosity, weaving/knitting patterns, etc.) and environmental conditions (air flow speed and direction, etc.) all influence the convective thermal regulation of fibers and textiles [65]. Simulation modeling can be helpful for understanding convective heat transfer processes and optimizing factors to enhance convective heat regulation capabilities [57].

**Figure 6.**
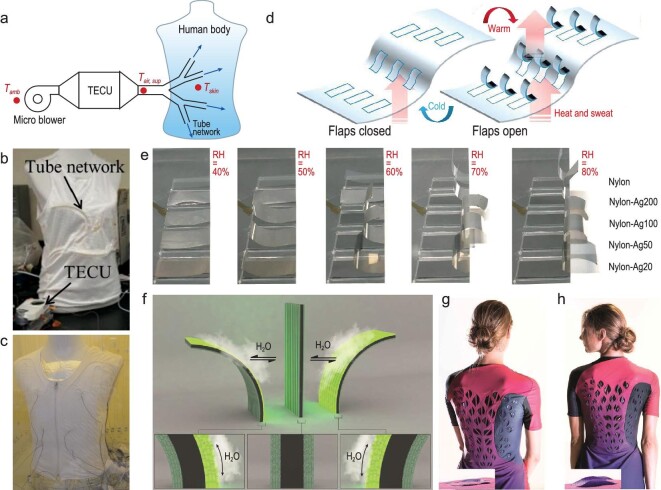
Convective thermal regulation with advanced textiles. (a and b) Schematic illustration (a) and photo (b) of the design of a tree-like tube network knitted into clothing for the purpose of heating or cooling via the TECU and micro blower. Reproduced with permission from [66]. Copyright 2018, Elsevier B.V. (c) Photo of the prototype of the LCG on the mannequin. Cooling tubes were sewn into the lightweight basic garment. Reproduced with permission from [68]. Copyright 2015, Elsevier B.V. (d) Schematic of the Nafion sheet with openable flaps for adaptive thermal regulation. Reproduced with permission from [63]. Copyright 2017, Nature Publishing Group. (e) Photos of the bending process of nylon-Ag actuators (length, 2 cm; width, 1 cm) in different humidities. Reproduced with permission from [69]. Copyright 2021, AAAS. (f) Shape transformation of a flat sandwich-structured biohybrid film when exposed to moisture. (g and h) Images of a garment prototype, (g) before exercise, with flat ventilation flaps, and (h) after exercise, with curved ventilation flaps. Reproduced with permission from [70]. Copyright 2017, AAAS.

For enhanced cooling or heating purposes, forced convection involving the active movement of air or liquid across the textile surface can be applied. Through integration of small fans, air pumps and fluidic devices within the textile design, forced convection systems can significantly improve the active heat exchange performance of textiles [66–68]. As depicted in Fig. 6a, Zhao *et al*. designed a tree-like rubber tube network that was knitted into an undergarment [66]. Cool or warm air can be blown out by a portable thermoelectric energy conversion unit (TECU) to provide satisfactory thermal comfort (Fig. 6b). Also, lightweight battery-powered fans were attached onto garments to increase forced ventilation for body cooling [66]. In addition to air convection, liquid cooled garments (LCGs) have been considered as a viable method to deliver a cooling effect through liquid coolant circulation in the flexible tubes embedded in the textiles [68] (Fig. 6c).

Dynamic responsive materials with flap ‘open’ and ‘close’ mechanisms can exhibit varying levels of convective heat dissipation, making them suitable for dynamic convective thermal regulation. These materials usually rely on moisture variation [63,69,70]. A Nafion film was cut to have openable flaps, whose inner-side-facing higher humidity can rapidly swell more than the outer portion. This mechanical deformation difference causes the flaps to bend upward and open up the pores, permitting more air flow [63] (Fig. 6d). Li *et al*. demonstrated a multimodal adaptive wearable with moisture-responsive flaps composed of a nylon/metal heterostructure (nylon-Ag) [69]. The bending angle can reach 260° when the local humidity below the nylon side increases from 40% to 80%, leading to a change of convective heat loss, as shown in Fig. 6e. It also exhibits great stability, showing an unchanged bending angle after 200 cycles [69]. By depositing genetically tractable microbes on a humidity-inert latex material to form a heterogeneous multilayered structure, Wang *et al*. obtained biohybrid films that can reversibly change shape within a few seconds in response to environmental humidity gradients [70] (Fig. 6f). They also demonstrated a running suit and a show prototype with bio-flaps that can dynamically modulate ventilation (Fig. 6g and h).

Materials that can respond to temperature or other external stimuli besides humidity by altering their structure or properties are also promising for dynamic convective thermal regulation. For example, shape memory polymers can change their configuration in response to heat, creating or closing air pathways within the textile [71,72]. Similarly, thermoresponsive hydrogels can expand or contract with temperature changes, modulating the airflow and heat dissipation properties of the fabric [73]. Integrating such materials into textiles allows for smart regulation of convective heat transfer, providing adaptive thermal-management solutions.

### Fibers and textiles for moisture-heat management

**Figure 7.**
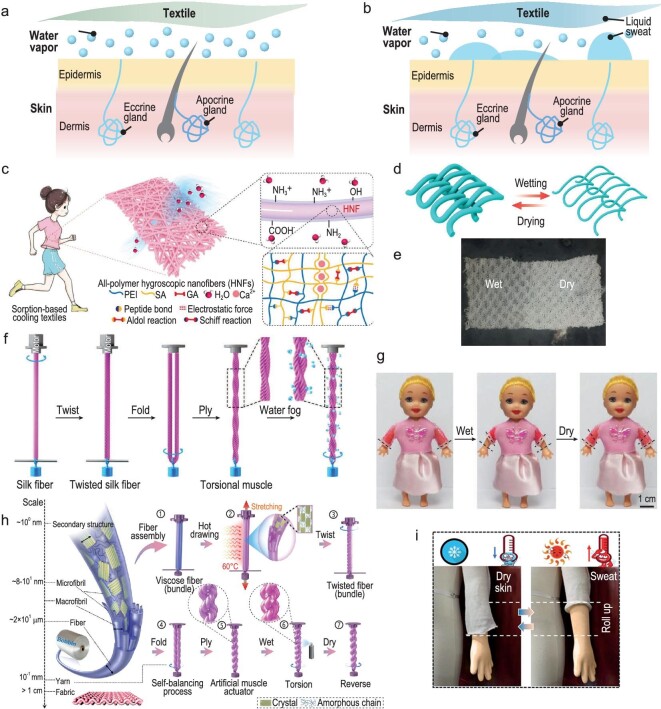
Human-body-evaporation heat-dissipation routes and fibers/textiles with advanced water-vapor regulation. (a and b) Schematic illustration of human-body insensible perspiration releasing water vapor (a) and liquid sweat secretion (b). (c) Working mechanism of the all-polymer hygroscopic fabric. Reproduced with permission from [78]. Copyright 2024, Wiley-VCH GmbH. (d) Schematic of the reversible loop-structure change during wetting and drying of the moisture-responsive yarns. Double-layer fabric structure consisting of two knitted layers, in which the outer layer is moisture responsive. (e) Photo displaying a comparison of the dry and wet parts of the bilayer cooling textile. (d and e) reproduced with permission from [79]. Copyright 2019, Springer. (f) Schematic illustration of the fabrication of a two-ply, torque-balanced silk torsional muscle. (g) Photos showing that the sleeves of smart clothing can contract when exposed to moisture and return to their original length when dry. (f and g) reproduced with permission from [71]. Copyright 2019, Wiley-VCH GmbH. (h) Hierarchical structures and fabrication process of viscose artificial muscles. (i) Sequence photos showing the sleeve of a garment made by viscose artificial muscles in a dry skin state (left) and a moisture-induced roll-up state (right). (h and i) reproduced with permission from [72]. Copyright 2021, American Chemical Society.

For the delicate human body system, with a narrow temperature operation range, evaporation plays an indispensable role in thermoregulation. At a mild state, ∼20% of human body heat dissipation relies on water vapor loss through insensible perspiration, as illustrated in Fig. 7a [74]. As heat load increases, liquid sweat evaporation must contribute to heat dissipation and even become the major heat dissipation route in intense scenarios (Fig. 7b), such as exercise and extreme environments [75]. Therefore, effective moisture management, including both water vapor and liquid sweat, is crucial for maintaining human comfort, health and performance.

#### Water-vapor moisture management

Decent water vapor permeability is one of the basic wearability requirements for clothing. Water vapor can be transported through textiles by the diffusion and adsorption/absorption-desorption process [76]. State-of-the-art textiles usually have a sufficient water vapor transmission rate to ensure comfort. On the other hand, different types of fibers display different hygroscopic behaviors [77]. For instance, cotton shows good absorption of moisture but is less efficient in quick release of moisture to the atmosphere. In contrast, synthetic materials, such as polyester, have relatively low absorption but better overall moisture-transfer ability. High-tech water-vapor-management textile materials are being studied and are attracting increasing attention, such as Gore-Tex technology. Gore-Tex is a patented, high-performance fabric technology recognized for its exceptional waterproof but breathable properties. Its core technology is the expanded ePTFE membrane containing microscopic pores which are ∼20 000 times smaller than a water droplet but larger than water vapor molecules. This unique structure prevents liquid water entry while permitting human-body water vapor to escape.

Sorption-based water-vapor moisture management is emerging. As shown in Fig. 7c, Li *et al*. developed 1D nanofibers and 2D fabrics using two hygroscopic polymers (sodium alginate and polyethyleneimine) and crosslinking strategies [78]. The designed fabric showed excellent hygroscopicity (equilibrium water uptake of 1.19 g g^−1^ in 4 h under 90% relative humidity) and high desorption rates, i.e. 80% of the absorbed water can be released within 10 minutes under mild heating. These high moisture sorption and desorption rates enable greatly accelerated moisture transfer from the human body to the environment [78].

Responsive textiles that switch between ‘open’ and ‘close’ states can dynamically regulate water vapor transmission. This regulation may overlap with the control of other heat dissipation pathways, as discussed in the works mentioned above [38,63,69–72]. In addition, Fu *et al*. designed a knitted fabric featuring a bilayer structure consisting of hydrophobic polyethylene terephthalate (PET) yarns inside, and moisture-responsive hydrophilic cellulose yarns outside [79]. The outside yarns changed from a loose to a dense structure after absorbing sweat, and the enlarged loop structure facilitated moisture and heat release (Fig. 7d and e) [79]. Another notable example is the woolen knitwear enabled with water-responsive switching of knit pores reported by Hu *et al.* [80]. They developed wool yarns with descaled fibers achieved through a special treatment and design. The fibers show two-way shape memory effect and lead to switching of the knit pores upon water stimulation [80]. Fifty consecutive cycles of shape-memory behavior in response to water have been demonstrated. This work is inspired, as it utilizes wool which is considered a natural thermal insulator for cooling applications.

An alternative strategy for dynamic moisture regulation is textile-length alteration through ‘artificial muscle’ polymers. Artificial muscles based on various kinds of materials, such as natural fibers, viscose fibers and lotus fibers, have been employed for moisture-responsive textiles. Thermally set, twisted, coiled and plied silk fibers were utilized as artificial muscles for weaving an actuation textile (Fig. 7f) [71]. The moisture absorption disrupted the hydrogen bonds of silk proteins, causing reversible expansion and contraction of the silk structure. As demonstrated in Fig. 7g, a 45% contraction for knitted sleeves in the wet state was demonstrated experimentally, and it returned to the original state under dry-body conditions [71]. Peng *et al*. reported a processing strategy to incorporate commercially available viscose fibers into artificial muscles with a hierarchical structure for moisture-responsive length-change textiles, as presented in Fig. 7h [72]. Based on the similar hydrogen bond disruption mechanism, the knitwear fabricated from the double-helical yarns can automatically roll up its sleeve in the wet state (Fig. 7i), enhancing heat dissipation and moisture escape from the human body [72].

#### Liquid-sweat moisture management

Apart from water vapor, liquid sweat is another form of human-body moisture. The interaction between textiles/fibers and liquid sweat is a critical factor in determining comfort and performance. The key mechanisms of their interaction involve wicking, absorption, desorption and evaporation [81,82]. Advanced fiber/textile technologies leverage these processes to effectively manage sweat, ensuring that sweat can be efficiently drawn away from skin, stored and eventually released, simultaneously maintaining thermal comfort.

The thickness, shape and hydrophilicity of fibers significantly influence the effectiveness of sweat transport in textiles [83]. Fine fibers, due to their smaller diameter, have a larger surface area, enhancing their ability to wick moisture through capillary action. This increased surface area allows for more efficient absorption and spreading of sweat. Additionally, the shape of the fibers plays an important role in moisture management. Fibers with unique cross-sectional shapes, such as grooves, channels, or multi-lobed structures, create pathways that assist capillary action and improve the transport of sweat. The inherent hydrophilicity or hydrophobicity of fibers also matters in sweat transport. Hydrophilic fibers attract and absorb moisture, helping to quickly draw sweat away from the skin. On the other hand, hydrophobic fibers repel water but can be engineered with hydrophilic surface treatments or combined with hydrophilic fibers to balance moisture management.

**Figure 8.**
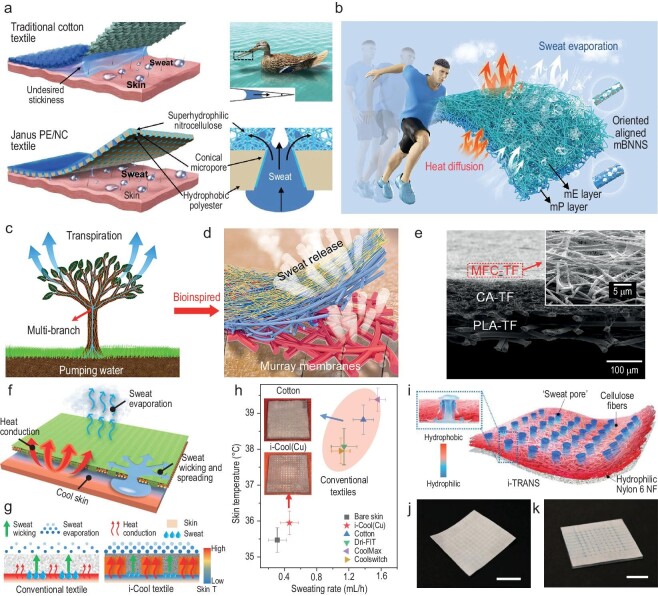
Textiles for liquid-sweat moisture-heat management. (a) Schematic illustration of the capillary force between a traditional cotton textile and wetted skin, and the sweat output pathways of the human body covered with the Janus PE/NC textile. Reproduced with permission from [84]. Copyright 2019, Wiley-VCH GmbH. (b) Schematic mechanism of heat diffusion and sweat evaporation in a DCT during the static and dynamic state of the human body. Reproduced with permission from [85]. Copyright 2024, Wiley-VCH GmbH. (c) The antigravity directional water transport of transpiration in nature. (d) Schematic demonstration of the sweat-release process of biomimetic porous Murray membranes. (e) Cross-sectional SEM images of the porous Murray membranes with trilayered architecture. (c–e) reproduced with permission from [86]. Copyright 2019, American Chemical Society. (f) Schematic of the working mechanism of the i-Cool textile. The water transport channels and heat conductive matrix work synergistically to efficiently evaporate sweat and cool down the human body. (g) Comparison between conventional textiles and the i-Cool textile. In contrast to normal textiles, the i-Cool textile can help the human body achieve an enhanced cooling effect with greatly reduced sweat, by using the sweat in a highly efficient manner. (h) Measurement results of skin temperature and sweating rate for bare skin, i-Cool (Cu), and commercial textile on an artificial skin platform with feedback control mimicking human body perspiration. Insets show the photographs of i-Cool (Cu) and cotton after 1-hour stabilization during the tests. (f–h) reproduced with permission from [81]. Copyright 2021, Nature Publishing Group. (i) Schematic of i-TRANS artificial sweating skin illustrating its structure and surface property. (j and k) Photographs of i-TRANS in a dry state (j) and wet state mimicking human skin perspiration (k). Scale bars, 1 cm. Reproduced with permission from [87]. Copyright 2022, Wiley-VCH GmbH.

Directional transport of sweat is challenging in conventional textiles, because they usually exhibit homogeneous wettability for the whole fabric. However, encouragingly, numerous studies have been done on developing textiles that can directionally transport liquid sweat. The key to achieving this lies in the construction of a wettability gradient across the textile cross-section direction [84–86]. Utilizing materials of different surface energy (hydrophilicity/hydrophobicity) to create a multilayered textile with anisotropic wettability is one effective strategy. As illustrated in Fig. 8a, Dai *et al*. demonstrated a hydrophobic/superhydrophilic Janus polyester/nitrocellulose (PE/NC) textile embedded with a conical micropore array with a hydrophilic inner surface [84]. This Janus textile can unidirectionally pump sweat from the inner hydrophobic surface to the superhydrophilic layer (a process driven by capillary force), with an ultrahigh directional water transport capability of 1246% [84]. Ding *et al*. designed a dual-cooling textile (DCT) that employs a Janus wetting structure for fast sweat evaporation, together with a 3D thermal conductive network for heat dissipation [85]. The fabric's outer layer is made of hydrophilic polyethylene-vinyl alcohol copolymer (EVOH) blended with modified boron nitride nanosheets (mBNNS), while the fabric's inner layer is made of hydrophobic polyurethane (PU) blended with mBNNS (Fig. 8b). Such a Janus structure endows the DCT with a transport index of 1081% and a fast water evaporation rate (0.34 g/h) [85]. Constructing a hierarchical structure composed of porous networks occupying different pore sizes is useful for realizing directional wicking. Wang *et al*. reported a biomimetic fibrous Murray membrane assembled by porous networks with across-thickness pore size and surface energy gradients, as shown in Fig. 8c–e [86]. It exhibited an ultrahigh one-way transport capability (R) of 1245%, an overall moisture-management capability (OMMC) of 0.94 and an outstanding water evaporation rate of 0.67 g/h (5.8 and 2.1 times higher than cotton fabric and CoolMax fabric, respectively) [86].

Furthermore, the thermal regulation of sweat evaporation is as important as optimizing its mass transport. Peng *et al*. focused on unlocking the cooling power of sweat evaporation and proposed an integrating cooling textile (i-Cool) for heat conduction and sweat transportation based on the laws of the human body perspiration mechanism [81]. The synergistic effect of heat conductive pathways and water transport channels makes i-Cool capable of evaporating sweat fast and cooling the body efficiently (Fig. 8f). A steady-state evaporation test revealed that i-Cool can achieve over 100% reduction in water mass gain ratio and three times the skin power density increment for every unit of sweat evaporation, compared to cotton [81]. Moreover, an artificial skin platform with a feedback control loop that simulates human body perspiration mechanisms was built. The i-Cool textile showed a 3–4°C lower skin temperature than traditional textiles with greatly reduced sweat consumption [81]. To better mimic sweating skin, the authors developed a facile surface modification method using poly(dimethylsiloxane) (PDMS) based on normal fibrous wicking materials for artificial sweating skin [87]. The integrated 3D hydrophilicity/hydrophobicity design is able to transport ‘sweat’ directionally without trapping undesired excess water and attain uniform ‘secretion’ of sweat droplets on the top surface, decently mimicking human skin perspiration [87].

Liquid-sweat moisture-management textiles are crucial for optimizing comfort and performance in challenging environments, and they do this by efficiently handling perspiration. A critical application of these textiles can be in military uniforms. The properties of directional sweat transport, being quick-drying, and having effective evaporative cooling guarantee optimal performance even during intense physical activities or in harsh climates.

## MANIPULATION OF HEAT STORAGE

The manipulation of heat storage by novel fibers and textiles has emerged as a thriving area of research and development, driven by its wide-ranging implications [88]. From enhancing personal comfort in everyday clothing to ensuring safety in extreme environments, the ability of textiles to store heat offers significant benefits. Also, textiles equipped with enhanced heat-storage properties play an important role in promoting energy saving and sustainability. For example, in building applications, integrating textiles with heat-storage capabilities into personal clothing, curtains and upholstery can significantly impact indoor climate control. By absorbing excess heat during warmer periods of the day and releasing it during cooler times, these textiles help maintain a stable indoor temperature without constant demand for artificial heating and cooling, thereby decreasing energy consumption [89].

Heat storage refers to the process of capturing and retaining thermal energy for later use. Specific heat capacity is a key physical quantity associated with heat-storage capability. In terms of endowing fibers and textiles with enhanced thermal storage capabilities, the integration of PCMs is still the most notable innovation [88,90]. Taking advantage of latent heat that can be stored or released from the PCMs as they transition between two states (usually solid and liquid states), PCM-integrated fibers and textiles can provide extra thermal-energy storage function. Diverse types of PCMs with transition temperatures in the range of 15°C to 35°C have been applied in fibers and textiles, including paraffins, hydrated inorganic salts and polyethylene glycol (PEG) [90].

**Figure 9.**
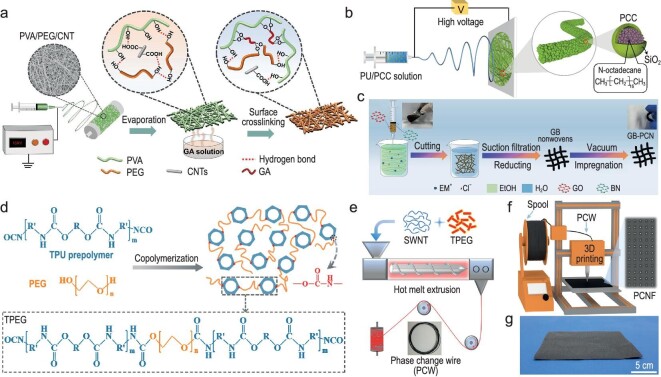
Fibers and textiles incorporated into PCMs. (a) Schematic illustration of the preparation procedure of eco-friendly phase change nanofibrous membranes. Reproduced with permission from [91]. Copyright 2022, American Chemical Society. (b) Schematic showing the fabrication and structure of FPU/PCC membranes. Reproduced with permission from [94]. Copyright 2019, American Chemical Society. (c) Schematic illustrating the fabrication of GB-PCN non-woven. Inset images are the photographs of graphene oxide-boron nitride (GO-BN) spinning solution (left) and GB-PCN (right). Reproduced with permission from [95]. Copyright 2023, Springer. (d) Preparation process of TPEG. (e and f) Melt spinning of PCW (e) and 3D printing of PCNF (f). (g) Photograph of a large-scale PCNF. (e–g) reproduced with permission from [96]. Copyright 2022, American Chemical Society.

Directly encapsulating PCMs into fibers is a way to prepare flexible PCM fibers. For example, as shown in Fig. 9a, Wu *et al*. filled biocompatible PEG into PVA fibers as a polymer skeleton by green electrospinning. CNTs were also introduced to improve thermal conductivity and tensile strength. Glutaraldehyde (GA) vapor was utilized to cross-link the polymer [91]. The prepared fiber-based membrane exhibited a reasonable temperature range (∼26.9–38.9°C) in the phase change process, with a melting latent heat of 60.1 J/g and freezing latent heat of 59.1 J/g as the mass ratio of PEG reached 55 wt% [91]. To prevent the material leakage when PCMs are converted into liquids, microcapsules/nanocapsules containing PCMs prepared by physical or chemical processes have been developed [92]. They can be incorporated into fibers. In Li *et al*.’s study, an n-eicosane microcapsule with a polyurea shell doped with copper oxide (CuO) was synthesized and applied to cotton fabric. The obtained fabric shows a heat capacity of 36.8 J/g, which is an improvement on that of the original fabric [93]. Yu *et al*. reported a type of corn-cob-like and phase-changeable nanofiber, which was incorporated with n-octadecane phase change capsules (PCCs) and terminal fluorinated polyurethane (FPU) [94]. The PCCs were uniformly distributed on the nanofibers (Fig. 9b), and the fabricated nanofibrous membranes presented high phase change enthalpy of 74 J/g even after 50 heating/cooling cycles [94]. Moreover, novel PCM host designs that are able to incorporate more PCMs to achieve high enthalpy have been explored. Liu *et al*. assembled a high-enthalpy flexible phase change non-woven (GB-PCN) by wet-spinning hybrid graphene-boron nitride (GB) fiber and then impregnating paraffins [95] (Fig. 9c). The abundant mesopores helped host paraffins, and the GB-PCN was shape-stable. The GB-PCN exhibited a very high enthalpy value of 206 J/g, anti-leakage capacity, exceptional thermal cycling ability of 97.6% after 1000 cycles, and showed ultrahigh water permeability. Wearable thermal-management systems (clothing and face mask) based on GB-PCN were demonstrated, and can maintain human body temperature at a comfortable range for an impressively long time [95].

Adopting solid-solid PCMs is another promising strategy for overcoming the high risks of liquid leakage of solid-liquid PCMs [96]. Polymers, such as olefin block copolymer, poly(vinyl chloride), poly(methyl methacrylate), TPU and polyethylene, are gaining traction due to their solid state during phase transitions [96]. Aiming at exploring and achieving facile and scalable manufacturing techniques for phase-change fabrics with multifunctionality, superior stability and durability, Yang *et al*. prepared a PEG-grafted TPU prepolymer (TPEG), as plotted in Fig. 9d, and blended it with single-walled carbon nanotubes (SWNTs) via hot-melt extrusion (Fig. 9e) to obtain phase-change wire (PCW). PCW can be continuously and efficiently printed out to build breathable phase-change non-woven fabric (PCNF) on a large scale, via 3D printing (Fig. 9f and g) [96]. The evenly dispersed SWNTs facilitated efficient heat harvesting and rapid transfer to solid-solid phase-change chains for heat storage. The PCNF exhibited excellent thermal reliability and stability with no leakage and shape variation after 800 cycles, and superior resistance to stretching/folding fatigue, without an obvious change in the mechanical robustness, conductivity and latent heat (65 J/g) even after 2000 cycles [96].

For PCM applications, low thermal conductivity can substantially impede the charge and discharge rates of PCM, which is one of the main challenges to be solved [97]. To overcome the low thermal conductivity of PCMs, the addition of thermally conductive agents has been widely investigated. Graphite, carbon fiber, carbon nanotubes and metal particles are all common additive materials for enhancing thermal conductivity [91,98,99]. Elgafy *et al*. studied the effect of carbon nanofiber additives on the thermal behavior of PCMs [98]. The thermal properties of modified PCM were enhanced significantly by dispersing carbon nanofibers into it. Chen *et al*. constructed a compactly interconnected 3D highly graphitized thermally conductive network via carbon quantum dots (CQDs) to infiltrate macromolecule PEG [99]. This strategy led to increased thermal conductivity (enhanced by 236%), and thermal enthalpy approached the theoretical value. By introducing CNTs into the electrospun fiber membrane of PVA-wrapped PEG, Wu *et al*. improved the thermal conductivity of resultant membranes by 40.4% with only 1.5% of the weight content of CNTs [91].

## MANIPULATION OF HEAT CONVERSION

In the realm of innovative fibers and textiles for thermal management, the manipulation of heat conversion complements manipulation of heat transport and storage, which represents another important avenue of development in this area. The manipulation of heat conversion for fibers and textiles refers to the strategic utilization of physical phenomena such as the photothermal effect, Joule heating and thermoelectric effect. These processes can be employed to realize controlled conversion of other forms of energy, like light and electricity, into heat, and transformation of redundant heat into electricity for reuse, enhancing thermal regulation capabilities for advanced textiles. Researchers and engineers have been exploring and utilizing these mechanisms to develop novel fibers and textiles. This section will focus on several heat conversion mechanisms and the corresponding material innovations in the context of thermal-management fibers and textiles.

### Photothermal conversion in fibers and textiles

The photothermal effect is a process by which certain materials convert absorbed light into heat. Three mechanisms of photothermal conversion have been proposed, namely, local heating of metal particles, non-radiative relaxation of semiconductors and thermal vibration of molecules, based on the interaction modes between electromagnetic radiation and matter [100]. This approach is valuable for providing localized heating without the need for external heating sources, making the technology attractive for various applications. The integration of the photothermal effect into fibers and textiles opens up new possibilities for directly harnessing sunlight to provide warmth, rendering it beneficial in outdoor and sports apparel, as well as therapeutic garments. Diverse types of solar-thermal conversion materials have been utilized for fibers and textiles, including metals, semiconductor materials, carbon-based materials, Mxenes and polymers [101–106]. The novel fibers and textiles are not only for personal thermal management, but also highly advantageous for solar steam generation.

**Figure 10.**
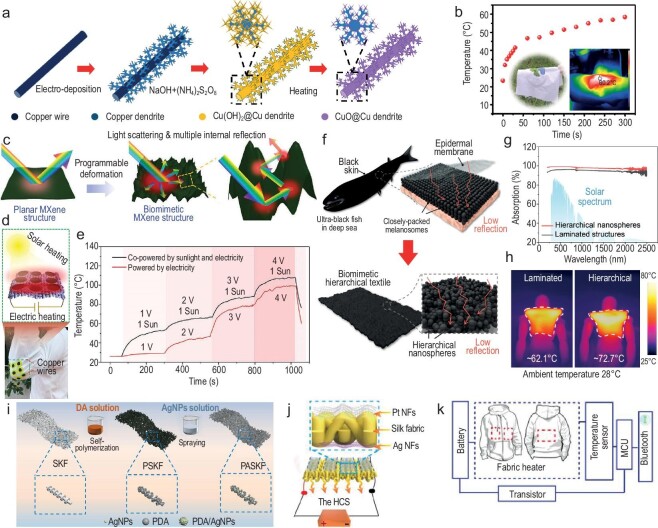
Fibers and textiles utilizing photothermal conversion and Joule heating. (a) Schematic diagram of CuO@Cu fractal dendritic photothermal fiber-preparation process. (b) Temperature of photothermal fabric within 300 s under an infrared light source. (a and b) reproduced with permission from [103]. Copyright 2023, Wiley-VCH GmbH. (c) Schematic illustration of the biomimetic MXene nanocoating with broadband light absorption and enhanced light-to-heat performance. (d) Schematic illustration (top) and photograph (bottom) show that the stretchable solar/electric MXene heaters were integrated on a T-shirt for wearable thermal management. (e) Surface temperature profiles of the solar/electric MXene heaters co-powered by one-sun illumination and different voltages. (c–e) reproduced with permission from [105]. Copyright 2019, Wiley-VCH GmbH. (f) Schematic of the ultrablack fish with unique black skin composed of closely packed melanosomes, and illustration of a synthesized bio-inspired ultrablack textile equipped with hierarchical PPy nanospheres for enhanced light capture. (g) UV−Vis−IR absorption spectra of a laminated and sphere-like PPy layer. (h) IR images of a laminated and hierarchical PPy textile under one-sun illumination. (f–h) reproduced with permission from [107]. Copyright 2022, American Chemical Society. (i) Fabrication process of the polydopamine-Ag nanoparticle shish-kebab superstructure films. Reproduced with permission from [111]. Copyright 2023, American Chemical Society. (j) Integration of the Ag NFs/silk fabric heater and the Pt NF temperature-sensor arrays. (k) Circuit diagram of the thermal control system. (j and k) reproduced with permission from [113]. Copyright 2019, IOP Publishing.

Simayee *et al*. synthesized copper (Cu) plasmonic NPs through a green method using red sanders extraction as the reducing matrix [102]. The Cu NPs significantly enhanced sunlight absorption and presented evident heat localization effect. A solar evaporator with an evaporation rate of 1.73 kg·m^−2^·h^−1^ and ∼98% conversion efficiency with regard to solar steam generation was demonstrated [102]. Yang *et al*. reported a flexible photothermal fiber based on Cu fractal dendrites with abundant CuO nanowires [103]. Well-aligned Cu fractal dendrite fiber was electrodeposited without templates in an aqueous solution. Then, direct *in-situ* oxidation and calcination of Cu dendrites produced CuO@Cu fractal dendritic photothermal fibers (Fig. 10a), which were subsequently woven into a photothermal fabric. The temperature of the photothermal fabric can be adjusted in the range of 35–65°C by adjusting the number of photothermal fibers (Fig. 10b) [103].

Zhao and co-authors reported an integral solar-heating yarn comprised of CNT fibers and cotton yarns twisted together and processed via carbon black slurry, which was primarily proposed for constructing high-efficiency fabric evaporators [106]. This unique structure of hybrid yarns featured a pronounced gradient photothermal difference of 5°C between the CNT and cotton regions in the dry state [106]. Carbon black NPs serving as light absorbing components were encapsulated by Jin *et al* inside polymeric nanofibers to prepare a highly flexible and washable non-woven photothermal cloth [104]. The photothermal cloth with an optimized carbon loading showed 94% absorbance of the solar spectrum underwater [104].

2D materials, such as Mxenes, are of particular interest in light-to-heat conversion. Researchers have developed facile methods to suppress their light reflection, as well as created prototypes for their practical application [105]. Inspired by the black scales of *Bitis rhinoceros*, Li *et al*. contrived a sequential thermal actuation approach to construct biomimetic 2D-material nanocoatings, including Ti_3_C_2_T_x_ MXene, reduced graphene oxide (rGO) and molybdenum disulfide (MoS_2_), as elucidated in Fig. 10c [105]. The hierarchical MXene nanocoatings achieved broadband light absorption of up to 93.2%, and enhanced light-to-heat performance (equilibrium temperature of 65.4°C under one-sun illumination). Moreover, the mechanically deformed MXene structures enable the fabrication of stretchable and wearable heaters that can be dual-powered by sunlight and electricity (Fig. 10d). They can be reversibly stretched and heated above 100°C (Fig. 10e) [105].

Additionally, polymers with a large number of π-electron delocalized structures have been applied for photothermal conversion, such as polydopamine (PDA), polyaniline (PANI) and polypyrrole (PPy) [107]. Xiao *et al*. were inspired by ultra-black deep-sea fish that have close-packed melanosomes. They developed a biomimetic ultrablack textile with the formation of hierarchical PPy nanospheres, as shown in Fig. 10f [107]. The close-packed nanosphere coating effectively captures the incident light, realizing even higher solar absorption than laminated structures, as exhibited in Fig. 10g. The authors demonstrated a photothermal vest based on the PPy textile with hierarchical structure. Compared to the laminated structure, the hierarchical structure exhibited a higher temperature of ∼72.7°C under one sun, as displayed in Fig. 10h [107].

### Joule heating fibers and textiles

Joule heating, also known as resistive heating, is a phenomenon whereby electrical energy converts into heat as an electric current passes through a conductive material. It has been extensively utilized in assorted application scenarios, among which is the field of fibers and textiles [108]. The Joule heating effect has been harnessed to develop advanced fibers and textiles that can provide well-controlled and efficient heating. It is often realized by modification or embedment of electrically conductive materials for fibers/textiles. Metallic materials, carbon-based materials and conductive polymers have all been reported as viable solutions for Joule-heating fibers and textiles [109]. In wearable technology, Joule-heating garments provide improved comfort and protection in cold environments, rendering them ideal for a range of applications. For outdoor enthusiasts, these garments help avoid the risk of hypothermia. In sports, athletes benefit from the consistent heat distribution, which can improve muscle elasticity and reduce injury risks. Military personnel equipped with these garments can operate more effectively in cold climates. In therapeutic settings, they assist in muscle relaxation and pain relief, offering a non-invasive method to support rehabilitation and recovery.

Zhao *et al*. fabricated a multifunctional MXene-based smart fabric by depositing Ti_3_C_2_T_x_ nanosheets onto cellulose fiber-based non-woven fabric (M-fabric) [110]. The metal-like conductivity of MXene endowed the fabric with an excellent Joule heating effect. It can serve as a low-voltage thermotherapy platform owing to its fast and stable electrothermal response, as well as its capacity to kill bacteria to a moderate extent, in the area surrounding a wound, in bacteria-infected wound-healing therapy. With an applied voltage of 6 V, the M-fabric reached a highest temperature of 100°C with a fast response time [110]. It also exhibited a sensitive and reversible humidity response upon water-induced swelling/contraction of channels between the MXene interlayers, making it suitable for application in wearable respiration monitoring. In addition, water molecular extraction can induce an electrical response upon heating, functioning as a temperature alarm, which could potentially help avoid burns [110]. A facile preparation method for the multifunctional wearable heater was developed by Xie *et al.*, based on Ag NP superstructure construction [111]. Using a shish-kebab superstructure film (SKF), melanin-like polydopamine was deposited, acting as an adhesive, on which Ag NPs were spray coated (Fig. 10i) [111]. This resulted in a wearable heater showing both enhanced photothermal conversion ability and active Joule heating (up to 72°C in ∼40 s at 0.6 V) [111]. Researchers have developed high-performance freestanding carbon-material-based wearable Joule-heaters. Guo *et al*. demonstrated an ultrathin graphene paper which is freestanding, flexible/foldable and wearable [112]. The superior electrical conductivity gives the excellent Joule heating effect, i.e. extra warmth of 42°C at a low supplied voltage around 3.2 V. Also, high durability during 500 bending cycles and a wash time of over 1500 minutes was revealed [112].

Integrating Joule-heating fabrics with a smart heating control system (HCS) is an appealing prospect. Huang *et al*. developed a sandwich-structural textile consisting of Ag nanofibers (NFs), silk fabric and platinum (Pt) NFs for an HCS [113]. The Ag NFs functioned as the Joule-heating components while the Pt NF network served as the temperature sensor, enabling simultaneous heating and temperature distribution detection (Fig. 10j). The conductivity and mechanical stability of the metal NFs were enhanced by crosslinking the free-standing fiber networks. With high thermostability, thermal resistance and temperature sensitivity, the HCS showed great potential in real-time wireless heating and temperature detection/control through a Bluetooth device in a smartphone [113]. As illustrated in Fig. 10k, a textile-based thermal controller system including a heater, temperature sensor, microcontroller unit (MCU) and Bluetooth module was designed. A desired temperature can be set by a mobile application, and a digital signal sent from the smartphone controls the power input for Ag NFs assisted by the temperature detection via Pt NFs [113].

### Fibers and textiles using thermoelectric effect

The thermoelectric (TE) effect, which involves the direct conversion of temperature differences into electrical voltage and vice versa, has promising applications in the field of fibers and textiles. By integrating TE materials into fibers and textiles, there is potential to create advanced fabrics that can utilize body heat or environmental temperature gradients to generate electricity or provide active heating/cooling by electricity input, implying various applications in energy harvesting and thermal regulation [114]. This innovative approach not only enhances the thermal functionality of textiles but also contributes to the development of sustainable and self-powered wearable technologies. Incorporating TE materials into fibers and textiles is significant but also challenging. The challenges include, but are not limited to, ensuring flexibility and durability of TE materials, optimizing manufacturing processes for scalability, and achieving efficient energy conversion. Addressing these issues requires innovations in material science, advanced manufacturing techniques and elaborate textile design.

**Figure 11.**
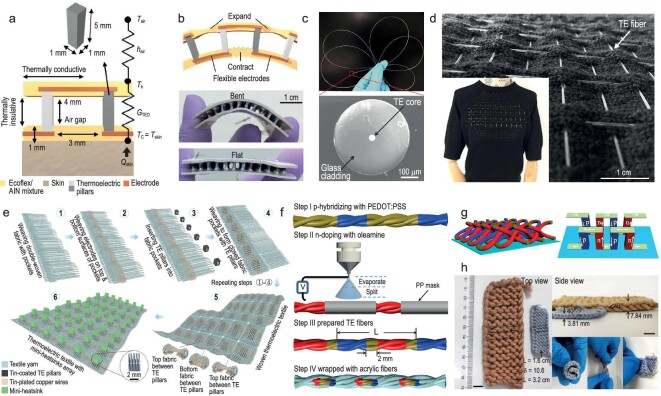
Thermoelectric fibers and textiles. (a) Schematic illustration of wearable-thermoelectric-device (TED) design. (b) Schematic diagram and photographs showing the flexibility of the TED. (a and b) reproduced with permission from [115]. Copyright 2019, AAAS. (c) Single TE fiber with a length of 1 m showing good flexibility, and its cross-sectional SEM image. (d) TE fibers are woven into a large area of fabric to construct a wearable TE device. (c and d) reproduced with permission from [116]. Copyright 2017, Elsevier B.V. (e) Schematic of the manufacturing process of the TET system. The insets in step 5 show the electrically in-series connections between the p-type and n-type TE pillars by tin-plated copper wires in the textile layers. Reproduced with permission from [117]. Copyright 2023, Royal Society of Chemistry. (f) Schematic illustration of the fabrication process of the TE yarns via doping CNT fibers. (g) Schematic illustrations of TE textile configuration. (h) Photographs of TEG textiles. (f–h) reproduced with permission from [14]. Copyright 2020, Nature Publishing Group.

The Peltier effect, a typical type of TE effect, reveals that heat can be absorbed or released at the junction of two different conductive materials, as an electric current passes through the junction. TE textiles can play a significant role in helping regulate the wearer's body temperature, providing both cooling and heating effects as needed. Zhao *et al*. proposed the use of a lightweight portable TE energy conversion unit to supply cooling or heating into the undergarment microclimate [66]. Lou *et al*. further optimized the integration with the clothing system and systematically evaluated its thermal performance [67]. They reported a novel lightweight (<1 kg) TE air conditioning undergarment system with a branching tubing network (branching angle of 60° and diameter ratio of 0.782) for air distribution. A maximum of 15.5 W of personal cooling and 18.1 W of personal heating with a coefficient of performance (COP) greater than 0.4 was demonstrated [67]. Hong *et al*. designed a flexible and wearable TE device (TED) using an innovative fabrication scheme of sandwiching rigid inorganic high-figure of merit (ZT) TE pillars between flexible elastomer sheets, as illustrated in Fig. 11a and b [115]. By virtue of their design, this device achieved a long-term (>8 hours) and large active cooling effect (>10°C) on the skin without the use of any heat sinks, with a high COP (>1.5) [115]. The flexible TED showed great potential with regard to personalized thermal comfort and lowering energy consumption.

Zhang *et al*. fabricated crystalline TE micro/nanowires by thermally drawing hermetically sealed high-quality inorganic TE materials in a flexible fiber structure, which exhibited the advantages of having high flexibility and mechanical stability, and being ultralong (Fig. 11c) [116]. The measured TE properties of the resulting p-type Bi_0.5_Sb_1.5_Te_3_ and n-type Bi_2_Se_3_ fibers showed that they had TE properties as high as their inorganic bulk counterparts. These TE fibers were woven into a large-area fabric, which can offer a maximum cooling effect of 4.9°C at a current of 3.5 mA (Fig. 11d) [116]. Recently, Jing *et al*. reported a scalable yet facile strategy to manufacture a large area (1550 cm^2^), durable, washable, skin-conformable and thus truly wearable TE textile (TET), by directly weaving inorganic TE pillars into the woven textile [117]. The fabrication process is exhibited in Fig. 11e. This material presented a rapid and stable body-surface cooling effect of ∼11.8 K and cooling capacity of ∼553.7 W/m^2^ under a breezy ambience of 34°C, with a maximum power consumption of 11.26 W in the TET [117].

In contrast to the Peltier effect that converts electricity into cooling or heating power, the Seebeck effect is the phenomenon where a voltage is generated across a conductor or a semiconductor when there is a temperature difference between its ends. Advanced fibers and textiles have been developed to harvest energy from body heat for electricity generation, which is valuable for developing self-sufficient wearable devices that do not rely on external power sources. Sun *et al*. made a TE fabric woven out of doped CNT fibers wrapped with acrylic fibers in π-shape [14]. The CNT fiber was p-hybridized by dipping into a commercial poly(3,4-ethylenedioxythiophene): poly(styrenesulfonate) (PEDOT: PSS) solution and n-type segments were fabricated at equal intervals by oleamine doping via the electrospray method, as exhibited in Fig. 11f [14]. Utilizing elasticity originating from interlocked TE modules, 3D TE generator (TEG) textiles with excellent stretchability (∼80% strain, with no output degradation) were fabricated (Fig. 11g–h), exhibiting a peak power density of 70 mW/m^2^ for a temperature difference of 44 K [14]. This work presented a truly wearable TE generator rivaling commercial cloth.

## CONCLUSION AND PERSPECTIVES

The field of thermal management using innovative fibers and textiles has experienced significant advancements. We are privileged to provide this brief but comprehensive review on the recent research advancements in fiber/textile material innovations that aim at enhanced thermal regulation properties. The regulation of heat was categorized into three aspects, namely, manipulation of heat transport, storage and conversion. In the section about heat transport, we divided the emerging works by their targeted heat transport pathways, including radiation, conduction, convection and moisture evaporation. Passive materials with improved performances and responsive materials with dynamic properties are both experiencing rapid growth and development. Novel fibers and textiles for heat storage are emerging too. Through hosting various kinds of PCMs in single fibers or fiber networks by delicate design, phase-change fibers and textiles are playing increasingly important roles in thermal management. Moreover, applications of energy conversion mechanisms involving thermal energy have been explored in fibers and textiles. In this article, we discussed the advanced fibers and textiles utilizing photothermal conversion, Joule heating and the TE effect.

The swift progress in this field is of paramount importance not only for promoting individual thermal comfort and personal efficiency but also for achieving significant energy savings. Additionally, these developments have promising implications for wearable electronics, where the integration with fibers/textiles and efficient thermal management are crucial factors. In the realm of defense security, innovative thermal-management textiles can enhance the comfort and effectiveness of protective clothing. Moreover, advanced fiber and textile materials are in demand for thermal regulation in the extreme conditions of outer space. Overall, the development of innovative thermal-management fibers and textiles presents a multifaceted approach to addressing both contemporary and future challenges. As research continues to push the boundaries of materials science and technology, we believe that thermal-management fibers and textiles promise to deliver more groundbreaking solutions that will impact numerous sectors positively and profoundly.

Regardless of the encouraging advancements, challenges still remain for further evolution of more cutting-edge fibers and textiles for thermal management. Here, we share several of our perspectives and look forward to inspiring more breakthroughs in this field.

First, for fibers and textiles used in personal thermal management, wearing comfort, safety and durability must be taken into account as a priority, as they are the most fundamental requirements for practical utilization. Comfort refers to the optimization of softness, breathability, stretchability and so on. The involved materials should be totally non-toxic and bio-compatible to prevent possible safety hazards. Also, improved longevity, so that the material can withstand wearing, tearing, washing and drying on multiple occasions is required. These properties, together with the thermal-management capability, ought to achieve a proper balance and a comprehensively decent performance for practical deployments.

Following this, the ability of thermal-management fibers and textiles to more aptly accommodate the specific needs of diverse scenarios is essential, for different scenarios expect customized features. To create multifunctional textiles that combine thermal management with other specifically tailored functionalities, such as antimicrobial properties, fire resistance, electromagnetic interference shielding and being bulletproof, is an effective strategy. Employing advanced materials science techniques and nanotechnology can optimize textiles for specific environmental and operational conditions. For example, the integration of conductive polymers and CNTs can enhance electromagnetic interference shielding, while the use of silica-based aerogels and aramid fibers can improve fire resistance and thermal stability. Additionally, embedding antimicrobial materials, such as silver NPs and copper oxides, can provide robust antimicrobial properties. We look forward to more advanced technologies for facilitating the development of multifunctional thermal-management textiles.

In the area of heat storage, future research should focus on exploring new and efficient PCMs. The objective is to enhance the durability and recyclability of PCM fibers, thereby improving their application lifecycle and reducing their environmental impact. The technical routes should include the development of novel composite materials that integrate PCMs with traditional fibers to achieve better thermal regulation properties while maintaining textile integrity and usability. Furthermore, to realize the full potential of these innovative fibers and textiles, it is vital to develop efficient methods for large-scale manufacturing. This involves creating scalable production processes that maintain high quality and consistency, thereby making these advanced materials more accessible and affordable for widespread use. Additionally, it is essential to invest in state-of-the-art manufacturing technologies that can handle the unique properties of these materials, such as advanced weaving techniques, precision cutting and automated quality control systems. Collaboration with industry partners may facilitate the integration of these methods into existing production lines, ensuring a smoother transition and faster adoption.

Moreover, the integration of thermal-management fibers and textiles with smart technologies and wearable electronics represents a promising frontier. As the demand for wearable technology continues to grow, thermal-management fibers and textiles can play a crucial role in enhancing the functionality and comfort of these devices. The innovation of fabrics that not only regulate temperature but also embed sensors, actuators and other electronic components, while self-generating power for their energy supply, could be a game changer. Meanwhile, the incorporation of smart technologies into responsive dynamic fibers and textiles could help us develop textiles that autonomously adjust their thermal properties, enabling the creation of highly responsive and versatile materials suitable for a wide range of application scenarios.

In addition, in the context of global warming, environmental sustainability is an increasingly important consideration in material development. Future efforts focusing on utilizing eco-friendly raw materials, reducing energy consumption during production, and ensuring that the end products are recyclable or biodegradable, will be beneficial. Innovations that enable textiles to efficiently harness and convert renewable energy sources, such as solar power and wind power, will be valuable.

In conclusion, the manipulation of heat transport, storage and conversion in fibers and textiles represents a remarkable frontier in materials science and engineering. By leveraging the unique properties of advanced materials and exploring innovative mechanisms, researchers and engineers have been creating next-generation fibers and textiles that meet the evolving demands of modern society. This field holds great promise for enhancing quality of life, improving energy efficiency and contributing to a smarter future. With continued innovation and interdisciplinary collaboration, the potential applications of thermal-management fibers and textiles are vast and varied, offering exciting opportunities for future advancements.
